# Epigenetic effects on the mouse mandible: common features and discrepancies in remodeling due to muscular dystrophy and response to food consistency

**DOI:** 10.1186/1471-2148-10-28

**Published:** 2010-01-27

**Authors:** Sabrina Renaud, Jean-Christophe Auffray, Sabine de la Porte

**Affiliations:** 1 Paléoenvironnements et Paléobiosphère, UMR5125, CNRS, Université Lyon 1, Campus de la Doua, 69622 Villeurbanne, France; 2 Institut des Sciences de l'Evolution, UMR 5554, CNRS, Université Montpellier 2, 34095 Montpellier, France; 3 CNRS, Institut de Neurobiologie Alfred Fessard, FRC2118, Laboratoire de Neurobiologie Cellulaire et Moléculaire, UPR 9040, Gif sur Yvette, F-91198, France

## Abstract

**Background:**

In wild populations phenotypic differentiation of skeletal structures is influenced by many factors including epigenetic interactions and plastic response to environmental influences, possibly blurring the expression of genetic differences. In contrast, laboratory animals provide the opportunity to separate environmental from genetic effects. The mouse mandible is particularly prone to such plastic variations because bone remodeling occurs late in postnatal ontogeny, in interaction with muscular loading. In order to understand the impact of this process on mandible morphology, we investigated how change in the masticatory function affects the mandible shape, and its pattern of variation. Breeding laboratory mice on food of different consistencies mimicked a natural variation in feeding ecology, whereas mice affected by the murine analogue of the Duchenne muscular dystrophy provided a case of pathological modification of the mastication process.

**Results:**

Food consistency as well as dystrophy caused significant shape changes in the mouse mandible. Further differences were observed between laboratory strains and between sexes within strains, muscular dystrophy causing the largest morphological change. The directions of the morphological changes due to food consistency and muscular dystrophy were discrepant, despite the fact that both are related to bone remodeling. In contrast, directions of greatest variance were comparable among most groups, and the direction of the change due to sexual dimorphism was parallel to the direction of main variance.

**Conclusions:**

Bone remodeling is confirmed as an important factor driving mandible shape differences, evidenced by differences due to both the consistency of the food ingested and muscular dystrophy. However, the resulting shape change will depend on how the masticatory function is affected. Muscular dystrophy caused shape changes distributed all over the mandible, all muscles being affected although possibly to a different degree. In contrast, the chewing function was mostly affected when the mice were fed on hard vs. soft food, whereas grinding likely occurred normally; accordingly, shape change was more localized. The direction of greatest variance, however, was remarkably comparable among groups, although we found a residual variance discarding age, sex, and food differences. This suggests that whatever the context in which bone remodeling occurs, some parts of the mandible such as the angular process are more prone to remodeling during late postnatal growth.

## Background

Identifying factors driving phenotypic differentiation in natural populations is often difficult because they are intricate and their effects are subtle. Genetic, developmental and environmental sources of variance interact to produce natural variation whose sources may include genetic differences, but also growth, sex, and effects of life-history traits such as the diet. In contrast, in laboratory models it is possible to control many effects and hence isolate given genetic or environmental sources of variance. The study of laboratory animals may thus help understand how a given cause can contribute to natural variations and evolution. Such inferences yet depend on to what extent laboratory cases mimic natural situations. Rodents and especially the house mouse are relevant to confront both aspects since their extensive phenotypic variation is documented in the wild (e.g. [1-3]) and also because the house mouse is one of the most common laboratory models, providing an extensive background on genetic, developmental and environmental effects on various aspects of its phenotype. The mandible of the house mouse was selected as the character of interest, because mandibles are obviously involved in the feeding process and hence prone to vary with the diet locally available to a wild population. This response is expected to be somewhat plastic because mandibles can be remodeled even during late postnatal growth by their interaction with masticatory muscles. The mandible also represents a well known model for evo-devo studies (e.g. [4-6]) that has contributed to evidence the complex, multigenic networks and developmental pathways underlying the observed patterns of phenotypic variance.

Some mutations isolated in laboratory strains have obvious effects that can pinpoint the implication of given genetic networks in morphogenesis. For instance, several mutations show the role of growth factors (e.g. [7-9]) or key developmental genes [10,11] in controlling the development of the skull and the skeleton. Such effects may be enlightening regarding genetic/developmental networks that might be recruited in macroevolutionary trends, e.g. tabby mice whose dental formula is reminiscent of mouse ancestors suggest that the EDA (ectodysplasin) signaling pathway is involved in the evolution of tooth formula in rodents [12]. However, such genetic changes unlikely contribute to the natural variation in wild populations because the dramatic phenotype alterations they cause would be lethal or strongly counter-selected in the wild.

Many mutations affect a given phenotypic trait in much more subtle ways, and this translates the fact that the mapping of genotypic into phenotypic effects is much more complex, involving more than just a few genes with major effects. Instead, phenotypic variations of the skull and mandible in the house mouse seem to be the result of many genes with partly redundant [5] and pleiotropic effects [4]. Furthermore, not all phenotypic changes relate to a change in the underlying genetic sequence, and the various mechanisms involved in such non-genetic changes occurring through various developmental processes are referred to as epigenetics. If today the term is often used in a narrow sense focusing on complex processes at the genomic level (e.g. [13,14]), it was coined to describe any aspect - other than DNA sequence - influencing the development of an organism [15] and under this meaning, it can refer to processes such as the influence one organ can exert on another during growth, e.g. mechanical effects of muscular loadings on bone growth. All these processes can contribute to the fact that a trait can vary, possibly in an adaptive manner, in response to biotic and/or abiotic environmental conditions, a characteristic known as phenotypic plasticity. Regarded as the ability of the bone to adapt to environmental clues, the changes in shape or bone density of the mandible under various muscular loadings can be regarded as plasticity [16], these changes occurring as the result of epigenetic interactions between muscles and bones.

Such effects are likely to contribute to the natural variations in the mouse mandible, for instance due to various diets among populations or along the year, requiring different masticatory strains and hence causing different loadings to be exerted on bones of the feeding apparatus. Because of the intricate processes underlying such plastic changes throughout postnatal growth, these effects are particularly difficult to disentangle in wild trapped animals [17]. It may be interesting to refer to laboratory models to assess how such processes affect the patterns of phenotypic variation. Indeed, some mutant mice display phenotypes that may mimic the patterns of plastic variations in wild populations. Mice with disturbed muscle growth may help understand how masticatory loadings affect the pattern of bone remodeling during growth and hence, can generate plastic changes in the mandible [16,18]. Such resulting patterns can be compared with those obtained by altering muscular loading, and hence bone remodeling, by feeding the animals on diets of various consistencies (e.g. [19,20]).

However, both procedures (differential muscle growth and food of different consistencies) may affect the mandible in different ways. Changes in diet could have targeted effects on muscles and zones directly related to chewing. In contrast, mutational changes in muscle activity could have much more global effects, affecting all muscles, related or not to food processing. Confronting both patterns of phenotypic changes should enlighten how plastic changes related to bone remodeling can act to shape the pattern of variance in a skeletal trait.

For such a purpose, we quantified the shape of the whole mandibular bone using a 2D outline analysis, confronting two sources of variation in muscular loading on the mandible: one mimicking a natural source of variation, namely diet, by breeding mice on food of various consistencies, and one related to a mutation not directly affecting mandible morphogenesis but the surrounding muscles: the murine X-linked muscular dystrophy (*mdx*), an analogue of the Duchenne muscular dystrophy in humans [21]. We compared the amount of morphological variance in these groups, the amount of phenotypic changes related to the different factors and the direction of morphological change in a morphometric space. We considered another aspect of the variance, namely the directions of greatest phenotypic variance. In natural populations they have been interpreted as "lines of least evolutionary resistance" because selection screens pre-existing variance and hence, response to selection is favored along directions displaying an important variation [22]. We investigated whether evaluating such directions was relevant in inbred laboratory strains where variance is reduced because of both inbreeding and controlled conditions, by estimating whether these directions were maintained in groups of laboratory mice, and if so, whether such directions of greatest intra-group variance matched the directions of change related to dystrophy or food consistency.

Significant differences between control and mutant mice, and between mice fed different types of food, would evidence an impact of muscular loading on patterns of variance via bone remodeling. A parallel between the directions of shape changes related to dystrophy and food consistency would further support the idea that bone remodeling occurred in the same way despite various causes of muscular loading differences. Finally, a parallel of those directions with the direction of greatest phenotypic variance in the laboratory groups would suggest that certain shape changes of the mandible occur preferentially in different contexts of bone remodeling. This would strongly evidence that this process is a major agent shaping the intra-population variance of the mouse mandible.

## Results

### Mandible size differences among groups

**Figure 1:**
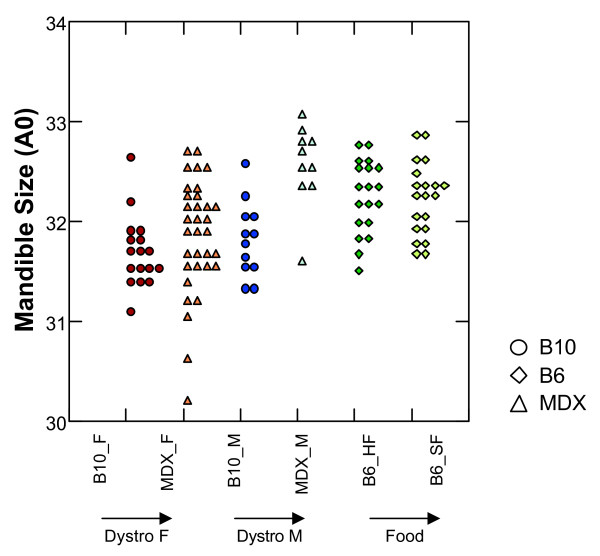
**Differences in mandible size, estimated by the zero harmonic of the outline analysis, between the B10 control and *mdx mice *(F: females; M: males), and B6 bred on food of different consistencies (HF: hard food, SF: soft food)**. Each dot corresponds to a mandible.

**Figure 2:**
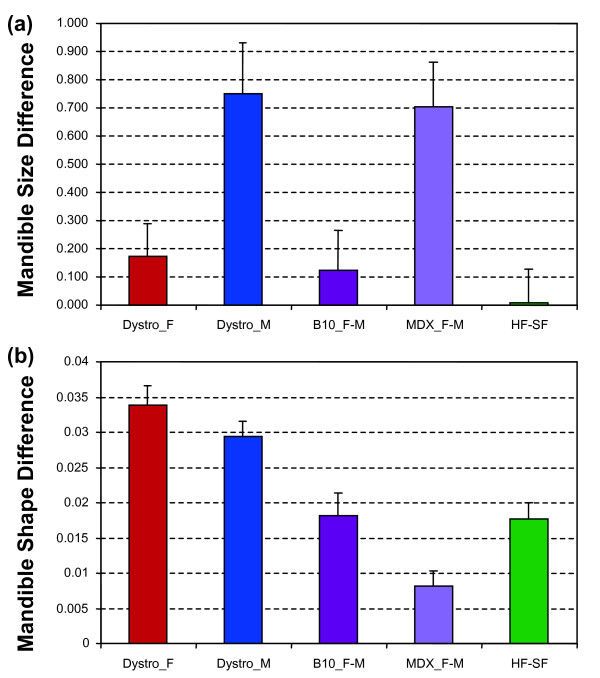
**Amount of inter-group difference in mandible size and shape between the B10 control and *mdx *mice for females (Dystro_F) and males (Dystro_M), between B10 males and females (B10_F-M) and *mdx *males and females (MDX_F-M), and between B6 bred on food of different consistencies (HF-SF)**. (a) Size. (b) Shape, the difference being estimated as the Euclidean distance between the average Fourier coefficients of each group. Error bars correspond to the standard deviation of the estimates based on 100 bootstraps of the initial samples.

Using A_0 _as size estimators, the effect of *mdx *muscular dystrophy appears to vary according to sex (Figs. 1, 2a): while control and dystrophic females do not significantly differ (*P *= 0.073), dystrophic males exhibit larger mandibles than controls (*P *< 0.001). Hence, while male and female control mice do not differ in mandible size (*P *= 0.352), dystrophic males are larger than females (*P *< 0.001). Response to food consistency in C57BL/6 mice does not cause any difference in mandible size (*P *= 0.937). Females of the B6 strain fed on regular rodent pellets were significantly larger than those of the B10 strain (*P *< 0.001).

### Amount of shape differences among groups

The strength of each effect was assessed as the length of the vector describing the difference between two groups (Fig. 2b) and tested using 2 by 2 multivariate tests. Significant shape differences emerge due to sexual dimorphism in C57BL/10 mice (*P *= 0.0008) and *mdx *mice (*P *= 0.0003). Mice fed hard vs. soft food differ in mandible shape (*P *< 0.0001). Dystrophy affects the mandible shape of females (*P *< 0.0001) and males (*P *< 0.0001). Finally females of the B6 and B10 strains fed on regular rodent pellets differ significantly in mandible shape (*P *< 0.0001). Regarding the amount of difference, the patterns are quite different for mandible size and shape. Food consistency affects shape but not size. Conversely, sexual dimorphism is particularly high in dystrophic mice for size but not for shape. Shape differences due to plastic response to food consistency and sexual dimorphism in B10 controls appear to be of similar magnitude, but two-fold lower than the dystrophic effect in males and females.

### Amount of size and shape variance

**Figure 3:**
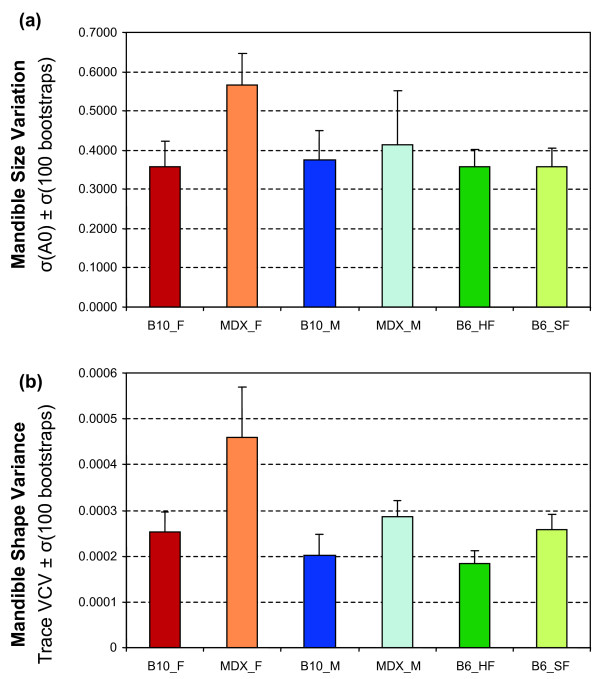
**Amount of within-group variance in mandible size and shape for the B10 control and *mdx *mice (F: females; M: males), and B6 bred on food of different consistencies (HF: hard food, SF: soft food)**. (a) Size variance, estimated as the standard deviation of the zero harmonic of the outline analysis. (b) Shape variance, estimated as the trace of the VCV matrices of the FCs (= sum of their variance). Error bars are the standard deviation of variance estimates based on 100 bootstraps of the initial samples.

The amount of shape variance also varies greatly depending on the group considered (Fig. 3). Conversely to the amount of difference between groups, the pattern of variance between groups is quite comparable for size (Fig. 3a) and shape (Fig. 3b). Less variable in size and shape are all the standard laboratory mice: C57BL/6 males and females and C57BL/10 females. Dystrophic females appear to be twice more variable in shape than the control groups. In contrast, dystrophic males display a variance close to the one of the control groups.

### Patterns of mandible shape differentiation

**Figure 4:**
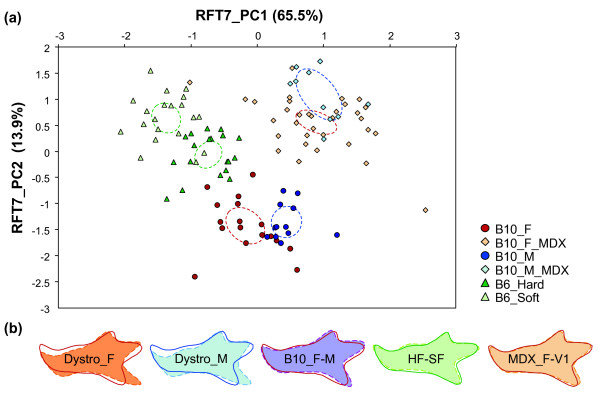
**Intra- and intergroup mandible shape variation between the B10 control and *mdx *mice (F: females; M: males), and B6 bred on food of different consistencies (H: hard, S: soft)**. (a) First principal plane of a PCA on the FCs of the mandible outline, including B10 control and *mdx *mice, and B6 mice fed on hard and soft diets. Each dot corresponds to a specimen; ellipses represent the 95% confidence interval around group mean. (b) Reconstructed outlines visualizing shape changes involved in some inter-group differences. From left to right: Dystro_F: change from B10 (full line) to *mdx *(dotted line, colored surface) in females; Dystro_M: change from B10 (full line) to *mdx *(dotted line, colored surface) in males; B10_ F-M: sexual dimorphism in B10 mice, from B10 females (full line) to B10 males (dotted line, colored surface); HF-SF: response to food consistency, from B6 on a hard diet (full line) to B6 on a soft diet (dotted line, colored surface). In all cases, the reference was the first group, and the change towards the second group was represented magnified three times. To the right: visualization of the direction of greatest phenotypic variation in the best sampled group (*mdx *females). The two outlines correspond to the average outline of the group ± three times V1. direction and length of V1 are arbitrary regarding the amount of change between groups. Outlines were reconstructed using an inverse Fourier transform, using EFT7 in order to provide accurate reconstructions; changes along V1 were estimated based on the coefficients of RFT7 and projected onto coefficients of the EFT7 using a multivariate regression.

The pattern of shape differentiation is summarized in the morphological space (Fig. 4a) corresponding to the first two axes of a PCA on the Fourier coefficients, totaling almost 80% of the variance. Along the first axis C57BL/6, C57BL/10, and *mdx *mice tend to segregate. The difference between *mdx *and C57BL/10 controls is even more clearly expressed along PC2, as well as the plastic response to food consistency in B6 mice and the B6/B10 difference. Among the differences between related groups, mandible shape changes caused by muscular dystrophy are almost twofold those related to sexual dimorphism in B10 mice, or to the response to food consistency in B6 mice.

Shape changes of the mandible (Fig. 4b) related to sexual dimorphism in B10 mice are mostly localized in the ascending ramus, with a more elongated angular process and dorsally shifted condylar and coronoid processes in males. Plastic changes in response to food consistency are localized in the posterior part of the mandible and especially the angular process, and in the zone of insertion of the molars that is slightly uplifted. In contrast, shape changes related to dystrophy are distributed all over the mandible in males and females. The most pronounced changes affect the angular process that is extended backwards, and the whole alveolar region that is shifted down.

### Directions of variation

Beyond the amount of difference involved, directions of morphological changes were compared (Table 1). Most vectors are robustly evaluated with less than an average of 15° between the original directions and bootstrapped vectors, except for sexual dimorphism in *mdx *mice, most probably due to the low sampling involved in the estimation of this vector (10 *mdx *males). Changes due to dystrophy in males and females are correlated, both corresponding to a downward shift of the symphyseal and molar alveolar region, as well as a backward elongated angular process (Fig. 4b). The direction of change from control to *mdx *females is further correlated to sexual dimorphism in C57BL/10, that also involves a lengthening of the angular process, but not to plastic changes due to food consistency, that involves an uplift of the molar alveolar region and a shortening of the angular process.

**Table 1:** Correlations between the vectors of mandible shape changes due to dystrophy, sexual dimorphism and response to food consistency.

	R_100_	θ_100_	Dystro F	Dystro M	Sex B10	Sex *mdx*	HF-SF B6
Dystro F	**0.991**	7.3°	-	36.7°	41.0°	64.8°	89.0°
Dystro M	**0.981**	10.5°	**0.802**	-	76.5°	36.4°	73.4°
Sex B10	**0.968**	13.1°	**0.755**	0.234	-	93.7°	58.2°
Sex *mdx*	**0.815**	33.8°	0.425	**0.805**	-0.065	-	85.7°
HF-SF B6	**0.972**	12.9°	-0.017	0.286	0.527	-0.075	-

First two columns: Average correlation (absolute value of *R *= inner product) and angle (Arccosine of |*R*|) between the original direction of change and its bootstrapped estimation (based on 100 bootstraps of the original groups). Next columns: Correlation (below the diagonal *R *= inner product and above the diagonal angle = arccosine of |*R*|) of vectors of changes due to muscular dystrophy in females (Dystro F) and males (Dystro M), sexual dimorphism in B10 mice (Sex B10) and dystrophic mice (sex *mdx*), and plastic response to food consistency in B6 mice (HF-SF B6). In bold significant probability (*P *> 0.99, |*R*| = 0.651).

The pattern of intra-group variance was compared among groups, by estimating the correlation between their directions of greatest phenotypic variance, namely the first eigenvector V1 of the VCV matrix. The reliability of their estimate varies greatly (Table 2) and is surprisingly not directly related to the number of specimens available. One of the smallest groups (B10 males, 12 specimens) provides the most stable estimate (8° of average angle between the original V1 vector and its bootstrap estimates), whereas the poorest estimate (30°) characterizes a relatively well-sampled group (B6 HF, 19 specimens). This is not due to the amount of variance (Fig. 3) since groups with similar variance level (B6 HF and B10 males) provide such contrasting results. It is rather due to the structure of the variance, B10 males being characterized by a variance with a clear major axis (V1 expressing 70% of the variance) whereas variance is more spherically distributed in groups such as B6 HF (V1 expressing less than 40% of the total variance).

**Table 2:** Correlations between directions of greatest intra-group variance.

	Nb	%V1	R_100_	θ_100_	V1 B10 F	V1 B10 M	V1 *mdx *F	V1 *mdx *M	V1 B6 HF	V1 B6 SF
V1 B10 F	17	58.0	**0.954**	15.9°	-					
V1 B10 M	12	61.6	**0.931**	17.2°	-0.288	-				
V1 mdx F	33	70.1	**0.988**	8.0°	**-0.893**	-0.084	-			
V1 *mdx *M	10	55.2	**0.923**	20.3°	**-0.892**	0.433	**0.820**	-		
V1 B6 HF	19	38.5	**0.819**	30.0°	0.365	0.642	-0.582	-0.096	-	
V1 B6 SF	20	52.0	**0.937**	17.8°	**-0.915**	0.539	**0.709**	**0.843**	-0.209	-

First two columns: Nb: number of animals in the considered group; %V1: % of variance explained by the first eigenvector of the VCV matrix between Fourier coefficients. Next two columns: Average correlation (absolute value of *R *= inner product) and angle (Arccosine of |*R*|) between the original V1 vector and its bootstrapped estimation (based on 100 bootstraps of the original groups). Next columns: Correlation |*R*| between V1 vectors describing major intra-group variation (+/- orientation of V1 vectors is arbitrary). In bold significant probability (*P *> 0.99, |*R*| = 0.651).

The V1 vector of B6 HF, with such a poor estimate, is not correlated with any major direction of variance of the other groups. The major direction of variance is correlated between B10 females, B6 on a soft diet, and dystrophic males and females. It mostly corresponds to a shortening/lengthening of the different processes, especially the angular process (Fig. 4b).

Mandible shape change linked to sexual dimorphism in B10 mice is related to the major direction of intra-group variance of B10 females, male and female *mdx *mice, and B6 on a soft diet (Table 3). The shape change due to food consistency is further related to V1 vectors of dystrophic females and B6 on a hard diet. Dystrophic changes are only related for females to V1 of B6 mice on a hard diet.

**Table 3:** Correlations between directions of greatest intra-group variance and directions of inter-group differences.

	V1 B10 F	V1 B10 M	V1 *mdx *F	V1 *mdx *M	V1 B6 HF	V1 B6 SF
Dystro F	-0.569	-0.125	0.610	0.343	**-0.667**	0.550
Dystro M	-0.034	-0.483	0.173	-0.223	-0.608	-0.027
Sex B10	**-0.912**	0.301	**0.811**	**0.785**	-0.443	**0.927**
Sex *mdx*	0.211	-0.558	-0.109	-0.485	-0.414	-0.323
HF-SF B6	-0.595	-0.346	**0.722**	0.441	**-0.708**	-0.075

Correlation |*R*| between V1 vectors describing major intra-group variation (+/- orientation of V1 vectors is arbitrary) and directions of changes due to muscular dystrophy in females (Dystro F) and males (Dystro M), sexual dimorphism in B10 mice (Sex B10) and dystrophic mice (sex *mdx*), and plastic response to food consistency in B6 mice (HF-SF B6). In bold significant probability (*P *> 0.99, |*R*| = 0.651).

## Discussion

The focus of the present study was to quantify and compare the phenotypic effect on the mouse mandible of two sources of plastic remodeling during postnatal growth related to muscle activity: differences in food consistency and pathological muscular defects due to dystrophy. We effectively found significant changes in mandible size and shape due to these factors, but also evidenced as corollary further differences between laboratory strains, between sexes, and interactions between sexual dimorphism and dystrophy, that make an overall intricate pattern of differentiation among the mandibles of these laboratory mice.

### Divergence between C57 Strains

The murine X-linked muscular dystrophy (*mdx*) spontaneously appeared in a strain of C57BL/10 inbred mice [23]; this strain was hence used as a model for the study of the dystrophic effect. This strain is however quite seldom used in experiments compared with the related C57BL/6J strain that we consequently used for experiments regarding food consistency. The analysis of mandible shape across the various groups evidenced a pronounced differentiation of these two strains. Significant divergence between mandibles of various strains has been previously documented (e.g. [24]) but such studies included distantly related strains, whereas C57BL/6J and C57BL/10 mice are genetically close [25], having been separated since about 1937. Yet, isolation and inbreeding can cause fast divergence in this fraction of time, as shown by insular population of mice achieving a significant differentiation in a similar amount of time [1]. Additionally, all these mice were females but of different age, the C57BL/10 ones being sacrificed at twelve weeks, and the C57BL/6J ones at 33 weeks, in order to provide a significant amount of time for remodeling in response to food consistency to accumulate its effects on the mandible. A part of the ageing might therefore contribute to the observed differences between the two strains, since mice at weaning have only reached about 70% of the degree of maturity for morphometric characters such as skull shape [26].

### Evidence of sexual dimorphism

A further and unexpected result evidenced by our study was the occurrence of a marked sexual dimorphism in mandible shape in C57BL/10 mice. It was unexpected because morphometric analyses on wild populations repeatedly suggested that sexual dimorphism was not significant in murine rodents for such characters (e.g. [2,27]). It may be that sexual dimorphism is not evidenced in wild populations because this effect is swamped out by other sources of variation, such as ageing and genetic differences. It may nevertheless contribute to the overall direction of greatest variance documented in wild mice and paralleling the directions of mandible remodeling occurring with late growth and response to food consistency [20].

The occurrence of sexual dimorphism in C57BL/10 mice might be related to such remodeling processes, because of behavioral differences between males and females. Males are more aggressive than females and aggressive mice from natural populations were suggested to display a larger insertion for muscles related to biting and attack [28]. However, the C57BL/10 strain is not characterized by high aggressiveness, with 10% of attacking males vs. 100% in some other strains [29]. It would be enlightening to investigate if sexual dimorphism in mandible shape is more pronounced in such aggressive strains, or whether it is due to other genetic or epigenetic factors.

### Plastic response to food consistency

The difference in mandible shape between mice bred on food of different consistencies evidenced a non-ambiguous case of plastic remodeling during the life of the animal. Mice from the same inbred strain were randomly split into two groups after weaning, one being fed with hard regular rodent pellets and the other with the same food under the form of jelly. The mandibles of the two groups clearly differed in shape after being bred for thirty weeks on their respective diets; the mice fed on a soft diet displayed a less robust alveolar region, a dorsally shifted molar alveolar region, and less robust angular processes. These changes are coherent with reduced loadings during the occlusion of the chewing molars, a movement driven by the masseter muscle inserted on the angular process. In contrast, no mandible size difference was recorded.

These observations were consistent with similar experiments on growing rats that were characterized by a thinner condylar process and a vertically reduced angular process together with a decrease in bone density [19,30]. These changes are due to bone remodeling of the mandible, a process that occurs along the entire animal's life, depending on the conditions of growth [19,30,31].

The magnitude of the mandible shape change related to food consistency was of the same order than sexual dimorphism in C57BL/10 mice. It was two-fold smaller, however, than shape differences related to dystrophy, despite a three-fold longer span for differences due to food consistency to accumulate.

### An important impact of the *mdx *dystrophy on mandible shape

The *mdx *muscular dystrophy is characterized by a complete absence of dystrophin that is involved in the maintenance of the morphological and functional structure of the striated, smooth and cardiac muscle fibers and in calcium homeostasis. Hence, no direct effect of this muscular dystrophy on bone morphogenesis is documented. However, the present study evidenced an important mandible shape difference between *mdx *mice and the controls. This effect is two-fold larger than other sources of differences between our laboratory groups, either sexual dimorphism, response to food consistency or divergence among strains. Mandible was globally affected, with modifications distributed all over it, but particularly marked in the alveolar region (where teeth are inserted) and in the angular process that is the zone of insertion of the masticatory muscles. Both zones are submitted to large strains during mastication and occlusion and bone remodeling was thus likely perturbed in *mdx *mice where deteriorated muscles cannot achieve the same force than in control mice. Yet, the fact that the effect of dystrophy is distributed all over the mandible is coherent with all muscles being affected, although possibly at various degrees [32]. This contrasts with the more localized shape differences caused by plastic response due to food consistency, focused on the parts of the mandible that are obviously involved in the masticatory process.

Qualitatively similar mandible shape changes involving "relatively longer and vertically shorter mandibles" have been reported in severely dystrophic C57BL/6J-*dy *mice [33]. The *dy *mutation is not sex-linked and causes more severe pathologies than the *mdx *one. The similarity in the phenotypic output of both dystrophies confirms that mandible shape changes are not directly related to the mutation but to the way deteriorated muscles, either due to the *mdx *or the *dy *mutation, interact with the bone during the development of the mandible.

The interpretation of the mandible differences due to *mdx *dystrophy is complicated by the occurrence of differences between males and females. Although the shape difference due to *mdx *dystrophy is parallel in males and females, shape differences appear larger in females in contrast to mandible size differences that are only marked in males. This interaction of sexual dimorphism and differential dystrophic effect leads to a significant dimorphism in size of *mdx *mice, but to the reduction of the dimorphism in shape compared with control mice. Such differences in *mdx *phenotypic outputs can be due to the fact that this mutation is sex-linked and might therefore differ in its effect between sexes [34]. Such subtle differences in the impact of the *mdx *dystrophy should be further investigated using larger samples, and at different ages since compensatory processes promoting muscle regeneration vary with age and sex [34].

### Patterns of variance in dystrophic mice and controls

Dystrophy not only affected mandible shape, but also increased the level of size and shape variance, especially in the best sampled group of *mdx *females. Deleterious mutations are thought to decrease canalization, i.e. the tendency for developmental systems to minimize the effects of genetic and environmental variations. Neither is the *mdx *mutation directly involved in the control of mandibular development, nor does it belong to the gene mutation types that promote canalization such as HSP90 [35]. Increased variance in *mdx *mice rather supports the view that canalization can be altered by various deleterious mutations, in agreement with similar results involving other mutations [36].

Despite this increase in the amount of variation, the direction of greatest phenotypic variance was parallel to the one characterizing other laboratory groups such as control C57BL/10 females and C57BL/6J females on a soft diet.

This homogeneity in the direction of variance among laboratory groups might is surprising since variance in these groups is residual, many sources of variance occurring in natural populations being discarded such as age, sex and genetic differences. The only residual source of variance should be individual differences in bone remodeling. How differences in muscular loading could affect the bone is very different in dystrophic or regular mice. However, some parts of the mandible such as the ascending ramus and especially the angular process appear to concentrate intra-group variation despite very different contexts. These parts are also affected by dystrophy and food consistency and do appear more prone to remodeling during late postnatal growth [6,18]. The directions of greatest variance in the laboratory groups may thus point to parts of the mandible prone to plastic changes: the ascending ramus, with thin processes where masticatory muscles insert, may vary more easily in response to muscular loading compared with the thick alveolar region reinforced by the internal part of the growing incisor.

### Different patterns of mandible remodeling

Bone remodeling has been advocated to contribute to various patterns of differentiation related to sexual dimorphism, plastic response to food consistency, and shape differences due to dystrophy. Yet, these different directions of shape changes are not all correlated one to the other. Dystrophy in males and females is correlated, an expected result since the same process is clearly involved. This "dystrophic" direction of mandible shape change is not correlated with the response to food consistency. Although the single process of bone remodeling might explain these different patterns, discrepancies might be explained by the fact that remodeling would occur in different contexts.

Regarding time, the different effects seem to be related to late postnatal growth, a period during which remodeling still occurs but at a slower rate and in different directions than pre-weaning growth [26]. Plastic response to food consistency occurred after weaning since the two groups were split from this time onward. Sexual dimorphism should likely develop in late growth as well, mice reaching sexual maturity at 5-7 weeks of age. Finally, the appearance of the pathological aspects between normal and *mdx *mice occurs after 2 weeks of the post natal life, but become really conspicuous after 3 weeks [37].

Alternatively, different functions and hence different muscles might be involved. The experiment with diets of different consistencies affected the process of chewing, and hence the molar zone and the zone of insertion of the masseter muscles, namely the angular process. It should not have affected grinding, and accordingly neither the incisor-bearing zone nor the coronoid process are involved in this kind of phenotypic response, two zones heavily affected when the function of the incisors is modified [38]. In contrast, muscular dystrophy does not affect all muscles at the same degree [32] but all are more or less affected. Hence, all functions of the mandibles, including both chewing and grinding, are likely affected by the disease, and this would explain why phenotypic changes related to dystrophy are distributed all over the mandible. Zones involved in changes related to aggressiveness seem to be more distributed, including masseter insertions but also lip muscles [28]. This case possibly provides an intermediate situation between a localized and a globally distributed response, exemplified by changes related to food consistency and dystrophy, respectively.

Such a globally distributed shape change does not seem to contribute much to the intra-group variation, since the direction of dystrophic change is poorly related to any direction of greatest variance. In contrast, response to food consistency and especially sexual dimorphism parallel the direction of greatest variance, even if such factors cannot contribute to the variation of unisex groups bred on the same food. It however suggests that non-genetic factors related to how muscular loading affect bone remodeling might contribute to the major patterns of intra-population variation. Remodeling, modulated by interactions with the muscles, still occurs late in post-natal ontogeny well after the developmental modules have been individualized, and may thus tend to modify the mandible as a whole [6,39]. The importance of such factors might explain why, despite the mouse mandible being a modular feature from a developmental and genetic point of view [4,5], shape changes characterizing microevolutionary processes involve the whole mandible instead to be localized on some modules (e.g. [6,20]). Changes in muscular loading related to food consistency would also be distributed because they recruit features involved in chewing across the modules of the mandible, namely the molars and the masseter. In contrast, modular response might be enhanced by contrasted mechanic properties of thin ascending ramus vs. the thicker alveolar region, as shown by patterns of greatest variance in the laboratory groups.

Overall, the study of non-genetic factors of phenotypic variation in response to differential muscular loading and mediated by bone remodeling during late postnatal growth appeared crucial to generate differences between groups but also directions of greatest phenotypic variance within groups. This direction is the first axis of the phenotypic variance-covariance matrix that is frequently considered as a surrogate of the genetic variance-covariance matrix, difficult or impossible to evaluate in wild trapped or fossil populations [40-42]. The present results however undermine this use, pinpointing the importance of non-genetic factors, including life-history traits and diet, and mediated by differential muscular loading and bone remodeling, as major agents contributing to the direction of greatest variance for a character like the mandible. In this case, the major direction of phenotypic variance appears rather as major direction of epigenetic variance, to which contribute various sources of plastic response of the mandible.

## Conclusions

The present study addressed the manner in which two potential sources of plastic shape variation affect mandible morphology: muscular dystrophy and food consistency. Neither one causes direct changes in the development of the mandibular bone but they both modify the muscular loading applied on the mandible. Both occur during late postnatal growth: mice were exposed to food of different consistencies after weaning, and the effects of the murine muscular dystrophy become obvious at about the same time. Both factors caused a clear shape change of the mandible, the muscular dystrophy causing a two-fold higher differentiation within a much shorter time span. Although both signals are related to bone remodeling, their morphological signature was different, muscular dystrophy causing a shape change distributed all over the mandible whereas the response to food consistency was more localized around the molar zone and the insertion of the masseter muscles. This suggests that despite offering elegant models, modifications due to mutations do not mimic processes occurring in wild populations. Muscular dystrophy affects all muscles, whereas differences related to food cause more targeted changes related to a given function of the mandible - chewing here - while other aspects such as grinding occur normally.

Despite these differences in the pattern of shape change achieved, the different groups shared similar directions of greatest variance. This shows that whatever the pattern of bone remodeling, some part of the mandibles are more prone to such changes. We further evidenced that several sources of shape differences may cumulate along this direction of greatest variance. Sexual dimorphism emerged as significant and the related direction of shape change paralleled the direction of greatest variance. Sexual dimorphism is usually not evidenced in wild populations where it is probably blurred by other sources of variation, such as ageing and food ingested. All these factors may cause bone remodeling during late growth and may cumulate along directions of greatest variance, corresponding to zones of the mandibles particularly prone to remodeling during late growth, partly masking variation of genetic origin in wild populations.

## Methods

### Dystrophic and control mice

Mice were bred at the Laboratoire de Neurobiologie Cellulaire et Moléculaire (Gif-sur-Yvette, France) with water and food (standard rodent pellets) *ad libidum *until the age of twelve weeks. The sample of dystrophic *mdx *mice included thirty-three females and ten males. Since the murine X-linked muscular dystrophy (*mdx*) appeared spontaneously in a strain of C57BL/10 inbred mice [23], this strain was used as control. Sampling included seventeen females and twelve males of the C57BL/10 (hereafter labeled as B10) bred in similar conditions than the *mdx *mice.

### Mice bred on different dietsx

Forty female mice from the inbred strain C56BL/6J (thereafter labeled as B6) were ordered at Charles River Laboratory and received when 3 weeks old, just after weaning. They were bred thereafter at the PBES (Ecole Normale Supérieure de Lyon, France) in four cages where they were provided with water and food *ad libidum *until they reached the age of six months (33 weeks) when they were sacrificed. Females were chosen for convenience, since they can be bred together in cages.

At the beginning of the experiment the mice were randomly split into two groups. Half of the mice received the ordinary hard pellet diet (hard food group, HF). The other group (soft food group, SF) was fed a gelatinous food obtained by grinding the pellets to a powder which was then mixed with agar-agar, and hydrated when given to the mice. The two diets were supposed to have the same energetic value for the growing mice because agar-agar is indigestible [43] and its addition to the diet of growing rats provided similar growth curves than control diet [44]. The final sample size was 20 mice for the SF group and 19 for the HF one.

The protocol of breeding and sacrifice has been validated as the regular procedure in the breeding stations. Since the ingestion of food was not considered as harmful, no further validation by an ethic committee was required.

### Mandible outline analysis

The shape of the mandible was estimated by its outline, corresponding to the 2D projection of the hemi-mandible placed flat on its lingual side. As the incisors may be mobile and some molars missing, only the outline of the mandibular bone was considered. This outline provides a good description of the processes involved in the insertion of the masticatory muscles, as well as of the alveolar region carrying the cheek teeth and incisors. For each mandible, the coordinates of 64 points at equal curvilinear distance along the outline were extracted based on photographs using the image analyzing software Optimas v. 6.5.

A radial Fourier transform (RFT) was applied to the mandible outline [17]. From the *x,y*-coordinates of the points along the outline, a set of radii (i.e. distance of each point to the center of the outline) was calculated. This set was analyzed as a function of the cumulative distance along the outline using a Fourier method. The initial dataset is thus described by a sum of trigonometric functions of decreasing wavelength, the harmonics. Each harmonic is weighted by two Fourier coefficients (FC). The zero harmonic (A_0_) is proportional to the size of each outline and was used as size estimator thereafter. It was also used to standardize all other FC in order to eliminate isometric size effects and to concentrate on shape information only. Previous studies on wood mice showed that considering the FC of the first seven harmonics offered a good compromise between measurement error, information content and number of variables to be considered [17].

An alternative approach to analyze outline data is the Elliptic Fourier transform (EFT). This method is based on a separate Fourier decomposition of incremental changes along *x *and *y *as a function of the cumulative length along the outline [45]. Each harmonic corresponds to four coefficients: two for *x*, and two for *y*, defining an ellipse in the *xy*-plane. This method offers an excellent reconstruction of the mandible outline by the inverse Fourier transform, useful for visual inspection. The RFT, however, has the advantage of providing a minimal number of theoretically independent variables. Statistical analyses were therefore performed on the FCs of the RFT7, whereas EFT was used to provide reconstructed outlines. Calculations were done using home-made programming on Mathematica for the RFT and EFAwin for the EFT [46].

### Statistical analyses

Differences in size were investigated using non-parametric Kruskal-Wallis (KW) test, which controls the absence of differences between the centers of the groups; it corresponds to a non-parametric analogue of a one-way analysis of variance.

The shape of each outline was described by a set of 14 Fourier coefficients (seven harmonics per two FC). The patterns of shape differentiation were investigated using a principal component analysis (PCA) on the variance-covariance (VCV) matrix, a method that provides synthetic multivariate axes expressing the main directions of the total variance. Differences among groups were tested using multivariate analyses of variance (MANOVA).

The amount of size variance in each group was estimated as the variance in the zero harmonic A_0_. The shape variance was estimated as the trace (i.e. the sum of the diagonal elements) of the VCV matrix. Statistics were done using Systat v. 12 and NT-sys v. 2.2.

### Comparison between P matrices and evolutionary directions

The matrices of phenotypic variance (**P**) were computed as VCV matrices based on the 14 FCs per mandible. Eigenvectors were extracted from VCV matrix for each group and normalized to unit length. The extraction of eigenvectors for such VCV preferably requires *ca*. 30 specimens to provide reliable estimates [47,48]; the robustness of the estimate nevertheless depends on the structure of the data [20]. Hence, **P **matrices were computed for all groups and their stability regarding sampling was assessed using bootstrapping. These eigenvectors were compared among groups as well as with the directions of differences between groups. These directions were also compared with one another. The shape change between two groups was expressed as the difference between the means of the FCs for each group.

The angle between two vectors is the arc cosine of the inner product of the two vector elements. Simulations of angles between random vectors were used to assess the statistical significance of this correlation [42]. Fifty thousand simulations of the correlation between two random vectors of 14 elements were performed. They provided the following significance thresholds for the absolute value of the inner product '*R*', probability that the observed *R *is higher than random: *P *> 0.95, *R *= 0.517; *P *> 0.99, *R *= 0.651; *P *> 0.999, *R *= 0.770; *P *> 0.9999, *R *= 0.860. Matrices computations were done using NT-sys v 2.2.

### Bootstrap procedures

A bootstrap procedure was used to estimate the robustness in estimating the direction of each vector. For the shape change Δ**z **between groups G_1 _and G_2_, each group G1 and G2 was bootstrapped 100 times, providing 100 vector differences that were compared with the original shape change Δ**z**. The robustness in estimating the eigenvectors of the **P **matrix was evaluated in bootstrapping each group 100 times. The corresponding VCV matrices and eigenvectors of the bootstrapped samples were compared with the original vectors. This procedure also allowed testing the robustness of the size and shape differences and variance estimates.

## Authors' contributions

SR designed the study, measured the samples, performed the statistical analyses and wrote the manuscript. JCA assisted in designing the study, contributed to the statistical analyses and to the preparation of the manuscript. SDLP provided all samples regarding dystrophic mice and their control, and contributed to the preparation of the manuscript.

All authors read and approved the final manuscript.
